# Cardiovascular events in CML patients treated with Nilotinib: validation of the HFA-ICOS baseline risk score

**DOI:** 10.1186/s40959-024-00245-x

**Published:** 2024-07-15

**Authors:** Fiona Fernando, Maria Sol Andres, Simone Claudiani, Nazanin Zounemat Kermani, Giulia Ceccarelli, Andrew J. Innes, Afzal Khan, Stuart D. Rosen, Jane F. Apperley, Alexander R. Lyon, Dragana Milojkovic

**Affiliations:** 1 Haematology department, Hammersmith Hospital, Imperial College Healthcare NHS Trust, London, W12 0HS UK; 2 Present Address: Cardio-Oncology Service, Royal Brompton Hospital, Guy’s and St Thomas’ NHS Foundation Trust, London, UK; 3 Centre for Haematology, Department of Immunology and Inflammation, Faculty of Medicine, Imperial College London, London, UK; 4 National Heart and Lung Institute, Imperial College London, London, UK

**Keywords:** Chronic myeloid leukaemia, TKI, Nilotinib, CV toxicity, Risk stratification

## Abstract

**Background:**

The therapeutic landscape of chronic myeloid leukaemia (CML) has been transformed by tyrosine kinase inhibitors (TKI). Nilotinib, showed higher rates of major molecular response than imatinib, however associated with higher cardiovascular (CV) toxicity. We sought to describe the CV events associated with nilotinib in a real-world population and assess the predictive value of the HFA-ICOS risk score.

**Methods:**

The HFA-ICOS baseline risk was calculated for patients with CML treated with nilotinib beween 2006 and 2021. The primary end point was the incidence of all CV events. The secondary end point was the incidence of ischaemic events. Survival analysis evaluated the risk (hazard ratio [HR]) of events stratified by baseline risk category, whilst on nilotinib therapy.

**Results:**

Two hundred and twenty-nine eligible patients were included. The incidence of CV events was 20.9% (95% CI: 15.7–26.2%) following a median duration of treatment of 34.4 months. The secondary end point occurred in 12.7% (95% CI: 8.4–16.9%) of the population. Patients with higher HFA-ICOS baseline score had higher rates of CV events (low: 11.2%, medium: 28.2% [HR: 2.51, 95% CI: 1.17–5.66], high/very high: 32.4% [HR: 3.57, 95% CI: 1.77–7.20]) and ischaemic events (low: 5.20%, medium: 17.9% [HR: 2.19, 95% CI: 0.97–4.96], high/very high: 21.6% [HR: 3.9, 95% CI: 1.91–7.89]). In patients who did not have a CV event, the median total dose at last follow up or cessation of nilotinib therapy was lower when compared to the total daily median dose of nilotinib in patients who had a CV event (450 mg vs. 600 mg, *p* = 0.0074).

**Conclusions:**

The HFA-ICOS risk stratification tool is an efficient discriminator at low, medium and high/very high risk of developing cardiovascular events, with an overall positive trend towards increasing cardiotoxicity rates with rising risk catergories. This study provides evidence to support the use of this predictive tool in nilotinib treated patients.

**Supplementary Information:**

The online version contains supplementary material available at 10.1186/s40959-024-00245-x.

## Introduction

Chronic Myeloid Leukaemia (CML) is a clonal haematopoietic stem cell disorder characterised by the pathognomonic reciprocal translocation t(9;22)q34 encoding the BCR::ABL1 fusion oncoprotein, which can be targeted therapeutically using tyrosine kinase inhibitors (TKI). BCR::ABL1 TKIs are standard of care for all patients with CML and have transformed their outcome, with most patients presenting in first chronic phase having a near normal life expectancy [1]. Nilotinib is a second generation BCR::ABL1 TKI, approved for first line and subsequent therapy, and shown to result in higher rates of major molecular response than the first generation TKI imatinib [2]. However, cardiovascular adverse events (CVAE), such as acute coronary syndrome, peripheral artery disease and ischaemic cerebrovascular disease have been reported at higher rates than for imatinib, thought to be as a result of the induction of hyperlipidaemia, effects on glucose metabolism and endothelial proliferation, upregulation of pro-atherogenic proteins, and off- target signalling [3]. The ENESTnd study assessed the longterm outcomes of front-line nilotinib vs. imatinib at 5 and 10 years, and significantly more CVAEs were seen in the nilotinib arm compared to imatinib. Five-year follow-up reported CVAE rates of 7.5%, 13.4% and 2.1% in patients on nilotinib 300 mg BD (twice a day), 400 mg BD and imatinib 400 mg OD (once a day) respectively [4]. Ten-year analysis revealed CVAE rates of 16.1% and 23.5% in patients on nilotinib 300 mg BD and 400 mg BD vs. 3.6% for imatinib^2^. An analysis of the FDA adverse event reporting system database reported high rates of CVAEs on TKI therapy, with 59% of the 3930 CVAE reported for TKIs related to nilotinib [5]. Hence, the benefits of nilotinib should be balanced against these potential risks. To date, the incidence of nilotinib-induced CV toxicity outside of clinical trials remains unclear as those recruited for clinical studies are often subjects with a lower burden of CV disease and comorbidities.

A subanalysis of the CML Study IV, a randomised trial designed to optimise imatinib therapy, showed that comorbidities at diagnosis have a negative impact on the overall survival of patients. CML is usually diagnosed at a median age of 60, increasing age coincides with an increase in prevalence of CV comorbidities [7]. CV toxicity may potentially impact on both morbidity and mortality of patients as well as lead to treatment interruptions and dose reductions, which then impact on overall disease control.

The ability to predict CV toxicity in those treated with nilotinb is of particular importance, as most will require lifelong daily treatment. The recent 2022 cardio-oncology guidelines from the European Society of Cardiology (ESC) include a class 1 recommendation to perform a CV risk assessment in patients receiving second and third generation BCR::ABL1 TKIs [7]. Risk prediction allows physicians to apply risk mitigating measures such as CV risk factor management and referral to a cardio-oncology service or consideration for another BCR::ABL1 TKI in high and very high CV risk patients. CV screening and management have also been incorporated into CML treatment guidelines [8–11]. In order to minimise the risk of CV toxicity, an appropriate CV baseline assessment is required, which is carried out inconsistently in many centres [12]. CV risk scores such as Framingham Score, the European Society of Cardiology (ESC) SCORE/SCORE-OP and British QRISK2 and JBS3 scores [14, 15] have not been designed nor validated in haemato-oncology populations. More recently, a predictive tool to calculate the baseline CV risk of patients commencing cardiotoxic therapies, including BCR::ABL1 TKIs has been developed and published by the Heart Failure Association (HFA) and the International Cardio-Oncology Society (ICOS) [16]. The use of this tool is recommended in the European Cardio-Oncology guidelines, however, the evidence regarding its effectiveness in a real world cohort is limited.

In this study we aim to describe the CVAEs associated with the use of nilotinib treatment in a single specialist centre and assess the performance of the HFA-ICOS CV risk prediction tool.

## Methods

We identified patients with CML treated with nilotinib between 15/11/2006 and 6/12/2021 at Imperial College Healthcare NHS Trust. Eligibility included patients with CML in any phase, treated with nilotinib for any duration of time. Predefined exclusion criteria included patients who were lost to follow up or had incomplete data sets due to treatment in other centres. Data regarding demographic characteristics, CV risk factors, disease phase, treatment doses and duration were gathered from the EPR. Data pertaining to baseline QTc intervals was unavailable.

Ethics approval for this analysis was attained by Imperial College Healthcare NHS Trust, as part of a service evaluation study. The study was exempt from collecting consent to participate, declarations from patients due to the observational nature of the analysis of data collected for routine clinical purposes, which was then analysed in an anonimised and retrospective manner. The STROBE guidelines and checklist for observational studies, were followed.

### Baseline Data Collection

Baseline patient characteristics prior to commencing nilotinib treatment were collected, including sex, date of birth, age at both diagnosis and beginning of treatment, Eastern Cooperative Oncology Group Performance Score (ECOG PS), comorbidities, smoking history, obesity, date of last follow up and date of death if applicable. CV history and risk factors were collected including known ischaemic heart disease, hypercholesterolaemia, diabetes mellitus, hypertension (HTN), arrhythmia history, cerebrovascular disease and chronic kidney disease. Baseline CML disease data were collected including date of CML diagnosis and date of nilotinib commencement, disease phase of CML, duration of nilotinib therapy, dose of nilotinib at initiation and cessation, line of therapy of nilotinib, molecular disease assessment prior to starting nilotinib and disease response measured by BCR::ABL1 RTqPCR on the international scale.

**Fig. 1:**
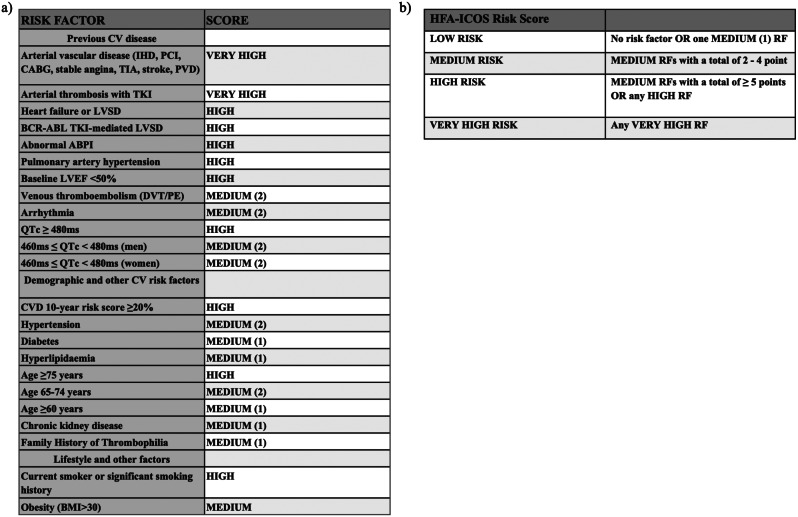
**a**. Risk factors and their corresponding score included in the baseline CV risk stratification proforma for BCR::ABL1 kinase inhibitors for chronic myeloid leukaemia including second and third generationTKI. BMI = Body mass index, CABG = Coronary artery bypass graft, IHD = Ischaemic heart disease, PCI = Percutaneous coronary intervention, PVD = Peripheral vascular disease, TIA = Transient ischaemic attack, LVSD = Left ventricular systolic dysfunction, CVD = Cardiovascular disease, AF = Atrial fibrillation, atrial flutter, ventricular tachycardia or ventricular fibrillation. **b**. Definition of each risk category according to the number of risk factors. RF = risk factor. Adapted from Eur J Heart Fail. 2020 Nov;22 (11):1945–1960. doi: 10.1002/ejhf.1920. Epub 2020 Aug 6. PMID: 32463967; PMCID: PMC8019326

The CV risk factors and co-morbidities of the patients were used to calculate the baseline risk of developing CV toxicity on nilotinib using the HFA-ICOS baseline risk stratification tool [16]. Patients were grouped into 3 categories according to the calculated risk: low, medium, high/very high (Fig. 1).

The study primary end point was a combination of all cardiovascular events during nilotinib treatment. A combined secondary endpoint was the incidence of ischaemic events during treatment with nilotinib defined as: acute coronary syndrome (ACS), cerebrovascular accident (CVA) and symptomatic peripheral vascular disease. The rate of subsequent CV events was assessed as a separate exploratory end-point. Only events that occurred while patients were taking nilotinib were included.

### Definitions

Cardiovascular event was defined as any acute disease affecting the cardiovascular system while the patient was receiving treatment with nilotinib. Acute coronary syndrome included unstable angina and acute myocardial infarction with or without ST segment elevation, as defined by the European Society of Cardiology guidelines [17]. Cerebrovascular accidents included transient ischaemic attack and stroke as defined by the American Heart and American Stroke Association guidelines [18]. Acute peripheral vascular disease was defined as new onset intermittent claudication or acute vascular occlusion with the need for interventional treatment. Subsequent CV event was considered in patients who developed a new acute problem in the cardiovascular system after the first event, while still under nilotinib treatment.

### Statistical analysis

Data were analysed with R studio Version 1.4.1717. Fisher’s exact test was applied for the analysis of all categorical variables. For continuous variables exhibiting a normal distribution (as determined by Shapiro-Wilk test), a t-test was applied. Variables with a non-normal distribution underwent analysis using the Mann-Whitney U test. A *P*-value of < 0.05 was considered statistically significant.

A multivariable logistic regression analysis including sex, age at nilotinib commencement, ECOG PS, time on nilotinib, nilotinib starting dose, smoking status, dyslipidaemia, obesity, known ischaemic heart disease, hypertension, chronic kidney disease and arrhythmia, was performed to assess for independent risk factors of CV events in this cohort.

Chi-square test for trend was conducted to assess for linear trends in the incidences of the primary and secondary endpoints across different risk categories.

Cox Proportional Hazard Ratios (HRs) and 95% Confidence Intervals (CIs) for the different risk categories were calculated to evaluate the relative risk of experiencing events in higher-risk categories compared to lower-risk. Stratification by HFA-ICOS score and adjustment for sex and ECOG PS were performed to control for potential confounding factors. Cox models were run separately for each end point. These models were compared with models with no adjustment for confounding factors using Akaike Information Criterion (AIC). The model with the lowest AIC was considered the best-fitting model, balancing goodness of fit with simplicity. Likelihood ratio tests were further conducted to compare the AICs of different models and ensure the chosen model adequately represented the data.

Time-to-event variables were analysed using the Kaplan–Meier method and were compared between groups using log-rank tests stratified by HFA-ICOS risk score. 95% CIs for Kaplan–Meier estimates were derived using the standard error calculated with Greenwood’s formula. Patients that did not have a cardiovascular event were censored at their last day on nilotinib or their last day of follow-up, whichever one occurred first.

Sensitivity, specificity, positive predictive value, and negative predictive value were computed to evaluate the predictive accuracy of the clinical prediction model. Additionally, the area under the receiver operating characteristic (ROC) curve was determined for all CV events and ischaemic events. An area under the curve (AUC) ranging from 0.5 to 0.59 was categorized as indicative of poor performance, while an AUC between 0.6 and 0.8 was considered acceptable, and an AUC above 0.8 was deemed excellent.

## Results

### Population characteristics

Two hundred and fifty-five patients started nilotinib between 15/11/2006 and 6/12/2021, of whom 26 were excluded after applying the predetermined exclusion criteria. Two hundred and twenty-nine (229) patients were eligible for analysis. The median age at nilotinib commencement was 49 years old (range: 19–90 years) and 49.7% of the patients were men. The CV risk factors and co-morbidities of the population are presented in Table 1.

**Table 1:** Demographic characteristics, CV risk factors and CML charcteristics of all patients and a comparison between those with and without a first CV event

	Total (*n* = 229)	No CV events (*n* = 181)	CV events (*n* = 48)	*p*-value
Age at Nilotinib commencement (median, range)	49 (19–90)	46 (19–90)	60 (25–77)	**< 0.001**
ECOG • 0 • 1 • 2	218 (95.1) 9 (3.9) 2 (0.87)	176 (97.2) 4 (2.2) 1 (0.55)	42 (87.5) 5 (10.4) 1 (2.1)	**0.013** **0.021** 0.376
Male (n,%)	114 (49.7)	84 (46.4)	30 (62.5)	0.052
HTN (n, %)	44 (19.2)	31 (17.1)	13 (27.1)	0.148
DM (n, %)	14 (6.1)	9 (4.97)	5 (10.42)	0.178
Dyslipidaemia (n,%)	107 (46.7)	78 (43.8)	29 (60.4)	0.051
Smoker or Ex smoker (n, %)	61 (26.6)	45 (24.9)	16 (33.3)	0.271
BMI > 30	50 (21.8)	42 (23.2)	8 (16.7)	0.327
Arrhythmia (n, %)	7 (3.6)	4 (2.21)	3 (6.25)	0.162
CKD (n, %)	11 (4.8)	5 (3.3)	6 (12.5)	**0.012**
History of ischaemic heart disease (n, %)	18 (7.86)	14 (7.73)	4 (8.33)	1.00
Disease phase at diagnosis: • Chronic • Blast • Accelerated	222 (96.9) 2 (0.8) 5 [1]	175 (96.7) 2 (1.1) 4 (2.2)	47 (98) 0 (0) 1 [1]	1 1 1
Nilotinib line • 1 • 2 • 3 • > 3	29 (12.7) 124 (54.1) 65 (28.3) 11 (4.8)	24 (13.3) 100 (55.2) 48 (26.5) 9 (4.97)	5 (10.4) 24 (50) 17 (35.4) 2 (4.12)	0.60 0.42 0.22 1
Total Months on Nilotinib (median, IQR)	34.4 (12.5–66.4)	34.5 (12.37–28)	34.4 (12.35–69.4)	0.15
Starting total daily dose of Nilotinib, mg (median, IQR)	600 (400–800)	600 (400–800)	600 (400–800)	1

HTN = hypertension, BMI = body mass index, CKD = chronic kidney disease

### Disease and treatment characteristics

The majority of patients treated were in chronic phase, with only 7 (3%) in accelerated or blast phase. Most patients were treated with nilotinib in the second line setting, with 12.7% of patients receiving nilotinib in first line. The median duration of treatment with nilotinib was 34.4 months (IQR: 12.35–66.4 months). The median initiation total daily dose was 600 mg (IQR: 400–800 mg) and the median follow-up time was 62.9 months (IQR: 38.49–91.23 months). Two hundred and eight patients (90.8%) were in at least complete cytogenetic response at their last follow up visit, however only 38% of those remained on nilotinib therapy.

### CV events and outcomes

**Fig. 2:**
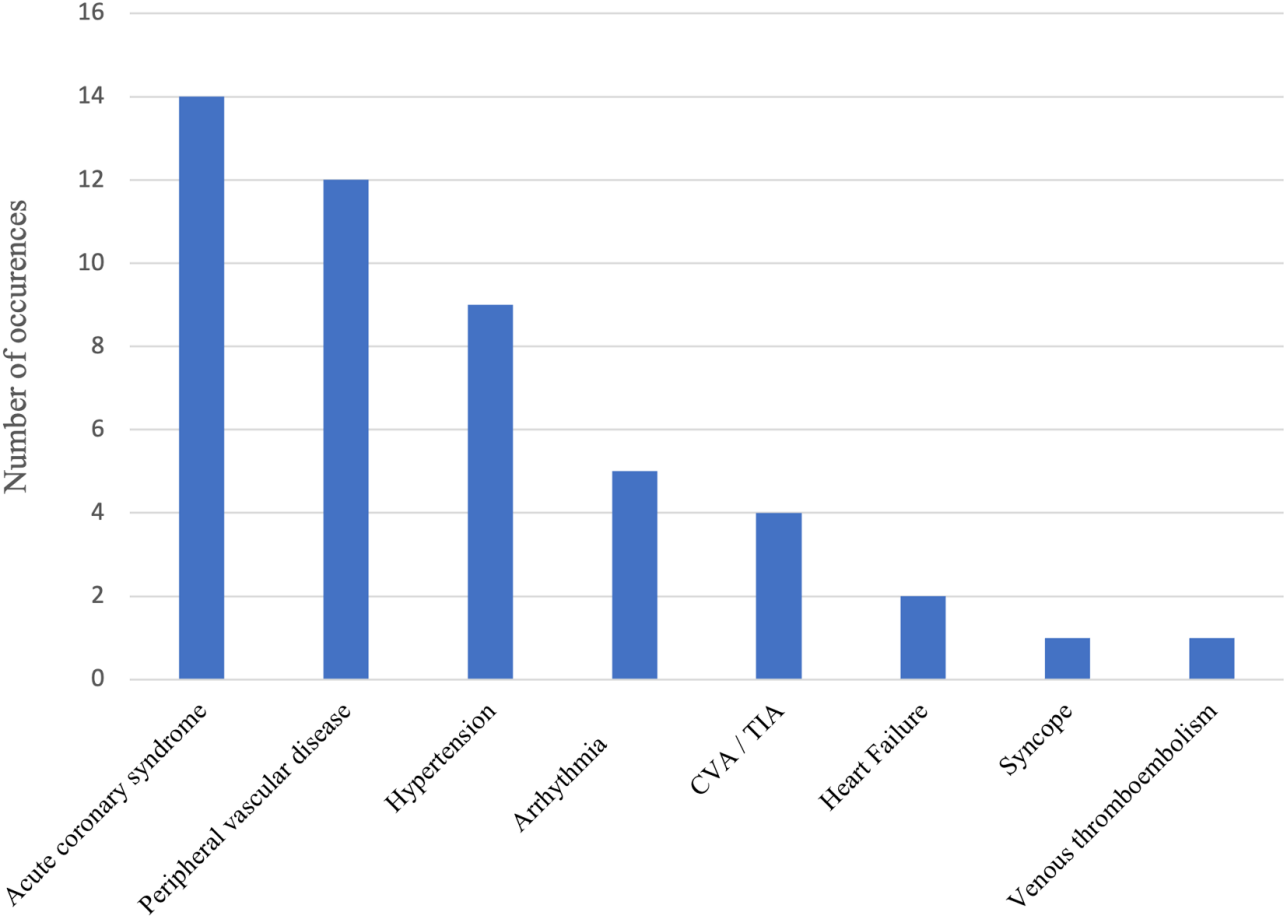
Frequency of cardiovascular events in CML patients receiving nilotinib. CVA = cerebrovascular accident. TIA = transient ischaemic accident. DVT/PE = deep vein thrombosis, pulmonary embolism

The primary end point, a combination of all cardiovascular events, occurred in 48 patients (20.9% [95% CI: 15.69–26.23%]). Fourteen patients (6.1%) presented with acute coronary syndrome, 12 (5.2%) patients had peripheral vascular disease and 9 (3.9%) developed new onset or worsening hypertension. Supraventricular arrhythmia including atrial fibrillation and paroxysmal atrial tachycardia occurred in 5 patients. Less common events included CVA (4/229), heart failure (2/229), syncope (1/229) and venous thromboembolism (1/229) (see Fig. 2). There were no deaths as a result of CV toxicity.

Those who experienced a CV event, were older in age and had a higher prevalence of chronic renal impairment. (Table 1). A multivariate logistic regression model identified age and male sex as independent factors associated with an increased risk of developing a CV event in this cohort (Table 2).

**Table 2:** Multivariable analysis to assess for independent cardiovascular risk factors associated with the development of a cardiovascular event

Predictor	Odds ratio	*p*-value	95% Confidence Interval
Sex (M - F)	2.85	0.014	1.25–6.63
Age at nilotinib commencement	1.05	< 0.001	1.02–1.09
ECOG: 1 − 0 2 − 0	4.7 1.19	0.067 0.91	0.89–24.8 0.05–23.9
Total months on Nilotinib	1.00	0.26	0.99–1.02
Nilotinib starting dose	0.46	1.00	0.99–1.00
Smoker (yes – no):	1.18	0.71	0.48–2.92
Dyslipidaemia (yes – no):	2.17	0.061	0.11–4.94
Obesity [BMI > 30] (yes-no):	0.57	0.27	0.21–1.54
Known ischaemic heart disease (yes – no):	0.49	0.35	0.11–2.12
Hypertension (yes – no)	0.76	0.62	0.25–2.26
Chronic kidney disease (yes – no)	6.5	0.057	0.52–33.4
Arrhythmia (yes – no)	4.20	0.18	0.44–13.93

M = male, F = female, BMI = body mass index

**Table 3:** Comparison of nilotinib total daily dose at the time of CV event against the total daily dose of nilotinib at the time of last follow-up or cessation in patients without a CV event

End points	No Event (mg)	CV event (mg)	*p*-value
Any CV event (median, IQR)	450 (400–600)	600 (437.5–800)	0.0074
Ischaemic event (median, IQR)	500 (400–600)	600 (600–800)	0.0118

CV = cardiovascular

In patients who did not have a CV event, the median total dose at last follow up or cessation of nilotinib therapy was lower when compared to the total daily median dose of nilotinib in patients who had a CV event (450 mg vs. 600 mg, *p* = 0.0074) (Table 3).

The secondary end point, a combination of ischaemic events (ACS, CVA and peripheral vascular disease) occurred in 12.7% (29/229 [95% CI: 8.36–16.97%]). Eleven patients had a subsequent CV event (4.8%, 95% CI: 2.03–7.57%) while on nilotinib treatment. The patients who developed more than one CV event were older at the time of diagnosis and nilotinib commencement but prevalence of other risk factors such as hypertension, diabetes mellitus and chronic kidney disease did not differ between groups (Appendix, Table 1). The proportion of patients with a poorer performance status (ECOG score ≥ 1) was higher among those who had multiple cardiac events (2.45% vs. 14.2%, *p* = 0.047).

### Performance of the HFA-ICOS risk prediction model

Upon utilisation of the HFA-ICOS baseline risk score calculator, our cohort of 229 patients was stratified as follows: 116 (50.7%) were categorised as low risk, 39 (17%) as medium risk and 74 (32%) as high/very high risk.

The rate of all CV events and ischaemic events positively correlated with an increasing HFA-ICOS baseline risk score. Specifically, the incidence of all CV events was 11.2% (95% CI: 5.5–16.9%) in the low-risk group, 28.2% (95% CI: 14.1–42.3%) in the medium risk and 32.4% (95% CI: 21.8–43.1%) in the high/very high risk category. A chi-squared test for trend confirmed the statistical significance of these disparities across the groups (*p* < 0.001). In terms of the risk of developing an event when compared against the low-risk category, after adjusting by confounders, the medium risk group showed a HR of 2.51, 95% CI:1.17–5.66, *p* = 0.026. The high/very high risk group presented a HR of 3.547, 95% CI: 1.77–7.20, *p* < 0.001.

Ischaemic events positively correlated with increasing risk categories: 5.2% (95% CI: 1.1–9.0%) for low risk, 17.9% (95% CI: 5.9–29.9%) for medium risk, and 21.6% (95% CI: 12.2–31.0%) in the high/very high-risk category, which was statistically significant (*p* = 0.002). The hazard ratio, contrasted against the low-risk group, after adjusting by sex and ECOG PS, was 2.19, 95% CI: 0.965–4.957, *p* = 0.060 for medium risk patients and 3.9, 95% CI: 1.91–7.89, *p* = 0.001 for high/very high risk patients.

**Fig. 3:**
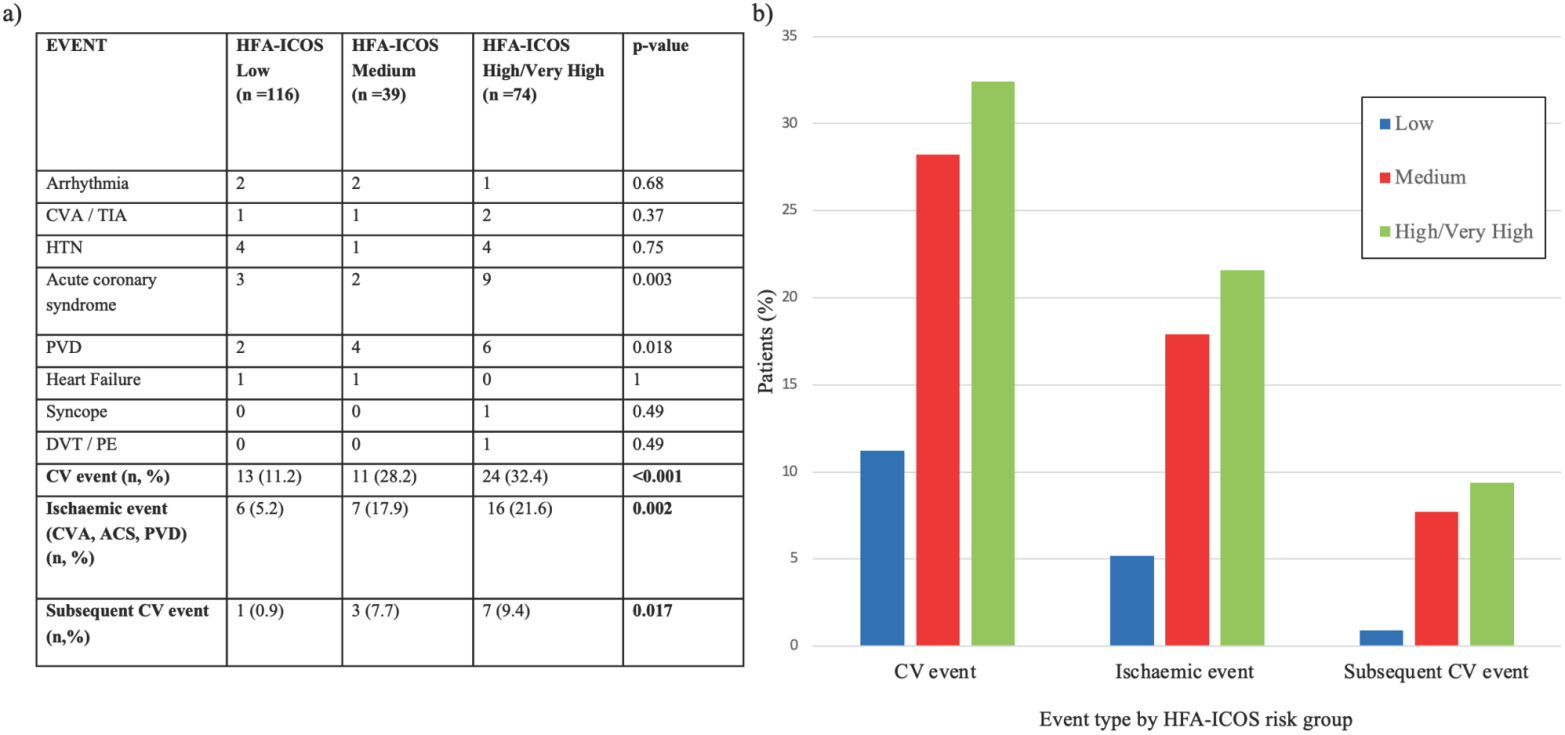
**a)** List of CV events and incidence of end-points, per HFA-ICOS risk score. **b)** Barchart showing the percentage of patients with a CV event, an Ischaemic Event and a Second CV Event grouped by baseline CV risk score. CVA = cerebrovascular accident, TIA = transient ischaemic accident, HTN = hypertension, PVD, peripheral vascular disease, DVT = deep vein thrombosis, PE = pulmonary embolism, MACE = major adverse cardiovascular event, ACS = acute coronary syndrome, CV = cardiovascular

Finally, the incidence of a second CV event was notably elevated in patients with higher baseline risk scores compared to their low-risk counterparts, as evidenced by the rates: low 0.86% (95% CI: 0.00–2.54%), medium 7.69% (95% CI: 0.00–16.06%) and high/very high 9.46% (95% CI: 2.79–16.13%), *p* = 0.017). These findings are shown in Fig. 3.

**Fig. 4:**
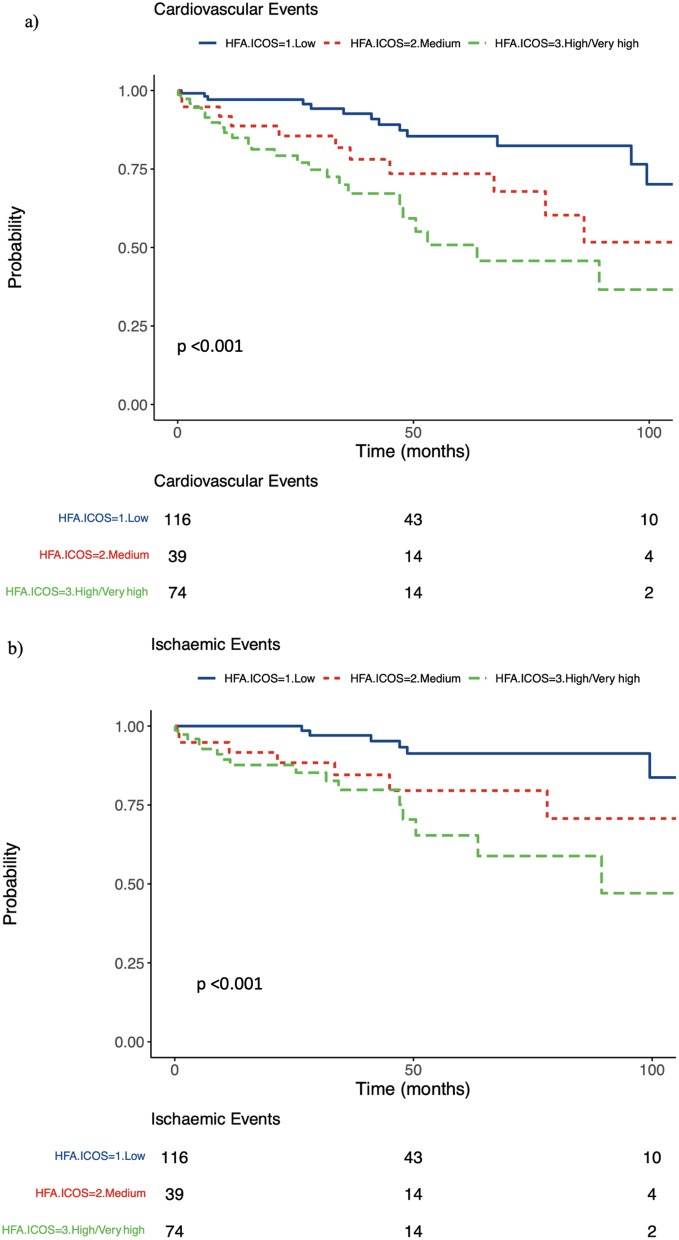
Kaplan Meier curves showing the time to event according to each risk category. (**A**) All cardiovascular events, (**B**) Ischaemic events

The median time to the first CV event and ischaemic events across all risk categories, was 31.7 months (IQR 9.36–48.2) and 28.2 months (IQR: 8.74–46.85) respectively. Kaplan Meier curves show a significant difference in time to event across risk group categories for the primary and secondary end points (Fig. 4)

**Fig. 5:**
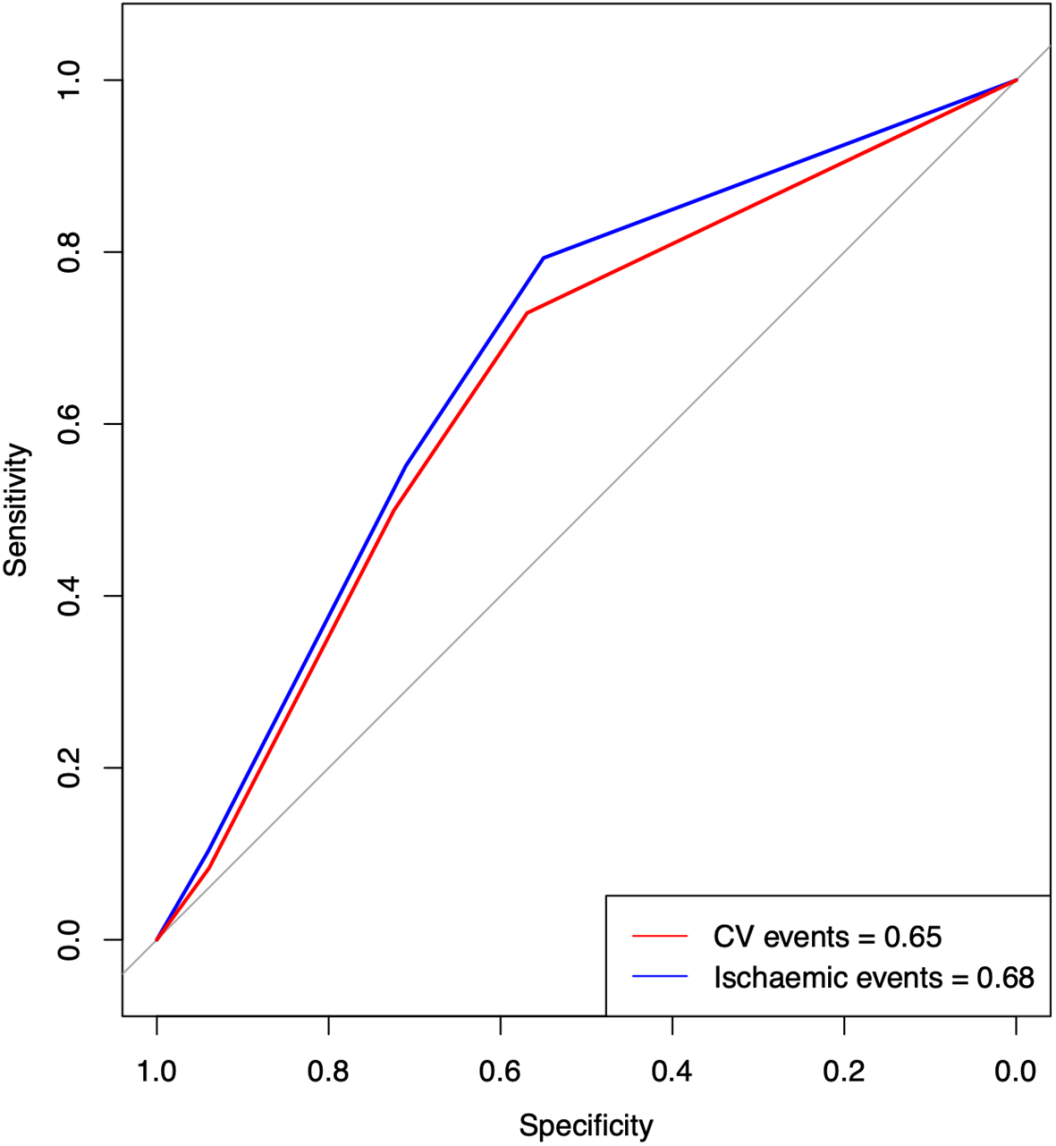
Receiver Operator Characteristic Curves to predict all CV events and Ischaemic events in patients on nilotinib using HFA-ICOS risk assessment tool. CV = cardiovascular

The HFA-ICOS Risk Tool performed with a sensitivity of 73%, a specificity of 55%, a positive predictive value (PPV) of 30% and a negative predictive value (NPV) of 89% to predict CV events occuring in patients classified as low risk against those classified as medium and high/very high risk. The area under the ROC curve for this predictive model was 0.65. The performance index observed for ischaemic events showed a sensitivity of 79%, a specificity of 54%, PPV of 19%, a NPV 95% and area under the ROC curve of 0.68 (Fig. 5).

## Discussion

CML is characterised by a personalised medicine approach and disease management differs to many other malignancies in that there is no single option for first line therapy. Consideration of several factors, including comorbidities which could be aggravated by a specific TKI, is crucial to the TKI selection process. Baseline cardiovascular risk assessment is a valuable and recommended strategy as it may mitigate the need for drug interruptions and importantly, avoid serious long-term cardiovascular disease. A recently published study showed that the HFA-ICOS risk score performs well in patients with CML treated with BCR::ABL1 TKIs, however the main limitations were that ischaemic events were the only reported CV events and the low number of patients included, resulting in a call for larger studies [19].

Here we present a large retrospective single-centre study reporting the incidence of nilotinib-induced CV toxicity in 229 patients with CML together with the assessment of the predictive power of the HFA-ICOS risk tool for CV events. The even distribution of HFA-ICOS risk catergories in this cohort, with 50.7% patients in the low risk and 49.3% in medium and high/very high risk categories allowed assessment and comparison of the rates of CV toxicity. Clinical trials of BCR::ABL1 TKIs routinely do not include high and very high risk cardiac patients as they often meet the study exclusion criteria, therefore this study provides valuable insights and is particularly informative for clinical practice.

The incidence of all CV events in this study was 21% across all risk groups, which is consistent with data from clinical trials, with reassuringly, no fatalities due to CV toxicity. Traditional CV risk factors such as hypertension, diabetes, dyslipidaemia and previous ischaemic heart disease, when considered individually, were not identified as independent risk factors of nilotinib induced cardiotoxicity. 50% of CML patients at the time of diagnosis present with CV risk factors, most commonly hypertension and diabetes as well as with pre-existing ischaemic heart disease [20] and our data suggests that nilotinib should not be excluded exclusively on the basis of a pre-existing cardiac risk factor or cardiac disease. However, the study only included 18 (7.9%) patients with pre-existing ischaemic heart disease, therefore small patient numbers may have impacted this finding. In contrast, when assessing a combination of all the risk factors using the HFA-ICOS risk tool, and adjusting for possible confounders, there was a clear positive correlation between the risk category and the development of the predetermined end-points including all CV events, ischaemic events and the rate of more than one CV event, underscoring the potential benefit of using this risk assessment tool, that combines the most relevant characteristics of a patient’s history, in contrast to estimating the risk based on independent risk factors or characteristics. This potentially suggests the preferential use of an alternative TKI over nilotinib, in selected cases, for those who are deemed high and very high risk. These results highlight the value of a baseline assessment and use of the HFA-ICOS risk tool to allow physicians select the most appropriate TKI.

There are some limitations to this study. We were unable to include QTc interval at baseline due to changing our EPR system, therefore physical ECGs were not available for review. For the purpose of the CV risk score calculation, this was assumed as normal and no events of torsade de pointes were recorded throughout the study. The patient number in the medium risk group is small, therefore analysis of this cohort may not be representative and has limited the ability of the HFA-ICOS prediction tool to discriminate between the medium and high risk patients. The lack of influence of CV risk factors when analysed individually was of interest, however this may be as a result of increased CV monitoring and concerted focus on CV risk reduction in the modern era of TKI management and the study was potentially undepowered to detect statistical differences between groups for each individual cardiovascular risk factor. Finally, these conclusions are derived from a single centre with retrospective data collection, which can be hampered by missing data, prone to both selection and patient recall bias which needs to be considered when interpreting the outcomes.

The study has a number of strengths, confirming that the incidence of CV events on nilotinib therapy remains clinically significant and is consistent with published clinical trial data. The predictive risk tool showed an 89–95% NPV for the primary and secondary end points, allowing robust identification of those at low risk of developing CV toxicity who would be able to start treatment without delay. The multivariable regression model identified male sex as being an independent risk factor for CV toxicity, this is particularly relevant as sex it is not included in the HFA-ICOS risk score, therefore the addition of sex to the prediction model could potentially improve its performance. Additionally, we demonstrated a dose dependent association of nilotinib and the development of any cardiovascular event, dose is not a parameter included in the baseline risk score assessment but should be considered when assessing the overall CV risk of the patient. This data suggests that while the HFA-ICOS baseline risk score has good sensitivity and high NPV, efforts may be directed towards improving specificity and refining the overall predictive performance. Prospective multicentre studies are needed for further validation of the score.

## Conclusion

In this single centre study, the rate of CV adverse events in adults with CML receiving nilotinib treatment is high, and the HFA-ICOS risk stratification tool has shown to be an efficient discriminator of CML patients at low, medium and high/very high risk of developing these events, with an overall positive trend towards increasing cardiotoxicity rates with higher risk catergories. The ESC guidelines now recommend baseline CV assessment in patients due to commence 2nd and 3rd generation TKIs in order to identify individuals who require intensive monitoring and treatment of CV risk factors with referral to cardiology, and cardio-oncology where available. These data provide evidence to support the use of this predictive tool in nilotinib treated patients and will inform and change clinical practice in accordance with the ESC recommendations.

### Electronic supplementary material

Below is the link to the electronic supplementary material.

Supplementary Material 1

## Data Availability

The dataset used for this study, with the anonymised patients’ data, is held by the authors and would be available for review upon request.

## Abbreviations

CML: Chronic Myeloid Leukaemia
HFA-ICOS: Heart Failure Associaion – International Cardio-Oncology Society
TKI: Tyrosine Kinase Inhibitor
FDA: U.S Food and Drug Administration
ESC: European Society of Cardiology
JBS3: The Joint British Societies for the prevention of cardiovascular disease
EPR: Electronic patient record
ECOG PS: Eastern Cooperative Oncology Group Performance Score
