# Supplementary material for: Cardiovascular events in CML patients treated with Nilotinib: validation of the HFA-ICOS baseline risk score
Source: Cardiooncology. 2024 Jul 15;10:42. doi: 10.1186/s40959-024-00245-x (PMC11247904; doi:10.1186/s40959-024-00245-x)
Supplement: Supplementary file 1 — Supplementary Material 1 [file 40959_2024_245_MOESM1_ESM.docx]

# Cardiovascular events in CML patients treated with nilotinib: validation of the HFA-

**ICOS baseline risk score**

**Appendix**

**Table 1. Demographic characteristics and CV risk factors patients with more than one CV event compared to patients with 0 to 1 CV events.**

|  | **0 to 1 CV events**  **(n = 218)** | **2 CV events**  **(n =11)** | **p-value** |
| --- | --- | --- | --- |
| **Age at diagnosis (mean, SD)** | 44.2 (14.8) | 54.7 (12.0) | **0.0001** |
| **Age at nilotinib commencement (mean, SD)** | 46.7(18.1) | 62.7(9.47) | **0.0009** |
| **Male (n,%)** | 108 (49.5) | 6 (54.5) | 0.77 |
| **HTN (n, %)** | 40 (18.3) | 4 (36.4) | 0.23 |
| **DM (n, %)** | 14 (6.42) | 0 (0) | 1 |
| **Dyslipidaemia (n,%)** | 101 (46.3) | 6 (54.5) | 0.76 |
| **Smoker or Ex smoker (n, %)** | 57 (26.1) | 4 (36.4) | 0.49 |
| **Obesity (n, %)** | 49 (22.5) | 1 (9.1) | 0.47 |
| **Arrhythmia (n, %)** | 6 (2.75) | 1 (9.1) | 0.29 |
| **CKD (n, %)** | 10 (4.58) | 1 (9.1) | 0.43 |
| **History of ischaemic heart disease** | 17 (6.4) | 1 (9.1) | 0.60 |

HTN = hypertension, DM = diabetes mellitus, CKD = chronic kidney disease.
